# Deep-learning-accelerated T1-MPRAGE MRI for quantification and visual grading of cerebral volume in memory loss patients

**DOI:** 10.1093/radadv/umaf022

**Published:** 2025-06-02

**Authors:** Nelson Gil, Azadeh Tabari, Dominik Nickel, Wei-Ching Lo, Bryan Clifford, Stephen Cauley, Min Lang, Sittaya Buathong, Azadeh Hajati, Shohei Fujita, Seonghwan Yee, John Conklin, Susie Huang

**Affiliations:** Department of Radiology, Massachusetts General Hospital, Harvard Medical School, Boston, MA 02114, United States; Department of Radiology, Massachusetts General Hospital, Harvard Medical School, Boston, MA 02114, United States; Siemens Healthineers AG, Forchheim 91301, Germany; Siemens Medical Solutions USA, Boston, MA 02110, United States; Siemens Medical Solutions USA, Boston, MA 02110, United States; Siemens Medical Solutions USA, Boston, MA 02110, United States; Department of Radiology, Massachusetts General Hospital, Harvard Medical School, Boston, MA 02114, United States; Department of Radiology, Massachusetts General Hospital, Harvard Medical School, Boston, MA 02114, United States; Department of Radiology, Massachusetts General Hospital, Harvard Medical School, Boston, MA 02114, United States; Department of Radiology, Massachusetts General Hospital, Harvard Medical School, Boston, MA 02114, United States; Department of Radiology, Massachusetts General Hospital, Harvard Medical School, Boston, MA 02114, United States; Department of Radiology, Massachusetts General Hospital, Harvard Medical School, Boston, MA 02114, United States; Department of Radiology, Massachusetts General Hospital, Harvard Medical School, Boston, MA 02114, United States

**Keywords:** brain, MRI, dementia, deep learning, MPRAGE, volume loss, volumetrics, variational network, super-resolution

## Abstract

**Purpose:**

To evaluate a physics-based deep-learning-accelerated super-resolution T1-weighted MPRAGE sequence (DL-MPRAGE) against standard 3-dimensional T1-weighted MPRAGE (STD-MPRAGE) for quantitative and qualitative regional cortical volume assessment.

**Materials and Methods:**

This prospective single-center study included patients undergoing evaluation for memory loss on 3T MRI scanners (MAGNETOM Vida, Siemens Healthineers, Forchheim, Germany) from October 2023 to January 2024. The absolute symmetrized percent change in cortical volume and thickness was assessed on DL- and STD-MPRAGE images using the FreeSurfer brain segmentation algorithm. Bland-Altman analysis evaluated the agreement in volumetrics for each anatomical region. Additionally, 2 blinded radiologists independently qualitatively rated image quality metrics and cortical volume loss for anatomical regions based on standardized scales.

**Results:**

A total of 64 participants (29 women [45%], mean age 62 years ±16 [SD]) were evaluated. DL-MPRAGE increased spatial resolution from 1 mm to 0.5 mm while reducing scan time by more than half (2:11 vs. 5:21). Mean regional volumes for DL-MPRAGE were systematically lower than for STD-MPRAGE (eg, 17 226 ± 2011 vs. 17 923 ± 2185 mm^3^, corresponding to an absolute difference between the means of 697 mm^3^, for the cingulate gyrus, *P < *.004). Corresponding absolute symmetrized percent change values averaged 2.8% across brain regions, with the largest mean value being 5.08% for the cingulate gyrus. Bland-Altman analysis demonstrated high agreement in quantitative measurements for both volume and thickness. On reader assessment, DL-MPRAGE was noninferior to STD-MPRAGE across image quality metrics (*P < *.01) and equivalent in assessing volume loss.

**Conclusions:**

DL-MPRAGE offers quantitatively and qualitatively equivalent volumetric estimation compared to STD-MPRAGE while improving spatial resolution and acquisition speed for patients undergoing evaluation for memory loss.

**Summary**

In brain MRI for memory loss, deep-learning-accelerated T1-MPRAGE, compared to standard T1-MPRAGE, offers qualitatively and quantitatively equivalent volumetric estimates, reduces acquisition time by more than half, and increases spatial resolution.

Key Results

- We prospectively evaluated a physics-based deep-learning-accelerated super-resolution T1-weighted MPRAGE MRI sequence (DL-MPRAGE) in 64 patients undergoing clinical evaluation for memory loss.
- The acquisition time of DL-MPRAGE is 40% that of STD-MPRAGE, and the axial spatial resolution is improved to 0.5 mm.
- DL-MPRAGE demonstrated noninferior image quality and no significant difference in quantitative or qualitative estimates of cerebral volume.

**Abbreviations**

MPRAGE: magnetization prepared rapid gradient echo, DL-MPRAGE: Deep-Learning Accelerated MPRAGE, STD-MPRAGE: Standard MPRAGE, MPRAGE: magnetization-prepared rapid gradient echo, TR: Repetition Time, TE: Echo Time, TI: Inversion Time, CAIPIRINHA: Controlled Aliasing In Parallel Imaging Results In Higher Acceleration, GRAPPA: Generalized Autocalibrating Partially Parallel Acquisition, ASPC: Absolute Symmetrized Percent Change

## Introduction

The clinical evaluation of dementia and other neurodegenerative diseases greatly benefits from high-resolution 3-dimensional (3D) neuroimaging for qualitative visual assessment. On the other hand, high-resolution neuroimaging is also crucial for quantitative volumetric estimation, especially in research settings.^1–3^ The standard of care for evaluating memory loss is based on brain MRI using 3D T1-weighted volumetric imaging such as magnetization-prepared rapid gradient echo (MPRAGE)^4^ due to its potential to provide superior spatial and contrast resolution of gray and white matter. However, evaluation of memory loss with MPRAGE can be limited by long acquisition times resulting in motion artifacts,^5^ which may be more common in clinical populations undergoing evaluation for memory loss.

Recent technical developments have improved upon the acquisition speed and reconstruction of high-resolution T1-weighted imaging sequences.^6–11^ Furthermore, although 1-mm isotropic resolution has been accepted as an adequate tradeoff between signal-to-noise ratio and acquisition time,^12^ submillimeter spatial resolution provides superior brain cortical surface reconstruction accuracy^13^ and has been adopted as a standard for consortia datasets,^14^ despite long acquisition times. A possible approach toward making submillimeter imaging clinically feasible is the use of deep learning (DL)-based image reconstruction and super-resolution algorithms,^15^ with a combination of variational network architectures to accelerate MRI acquisition,^16^ and convolutional neural networks to compute super-resolution images.

We introduce a physics-based DL-accelerated super-resolution T1-weighted MPRAGE sequence (DL-MPRAGE) that achieves up to a 0.5-mm resolution while halving acquisition time compared to standard MPRAGE. We clinically validate DL-MPRAGE by testing it in a head-to-head fashion against standard T1-weighted MPRAGE (STD-MPRAGE) on a prospective clinical cohort of patients with memory loss symptoms. We hypothesized that DL-MPRAGE would demonstrate equivalence in morphometric analysis compared to STD-MPRAGE and noninferiority in the qualitative assessment of regional cortical volume in patients undergoing clinical evaluation for memory loss.

## Methods

### Study design and imaging protocol

**Figure 1.**
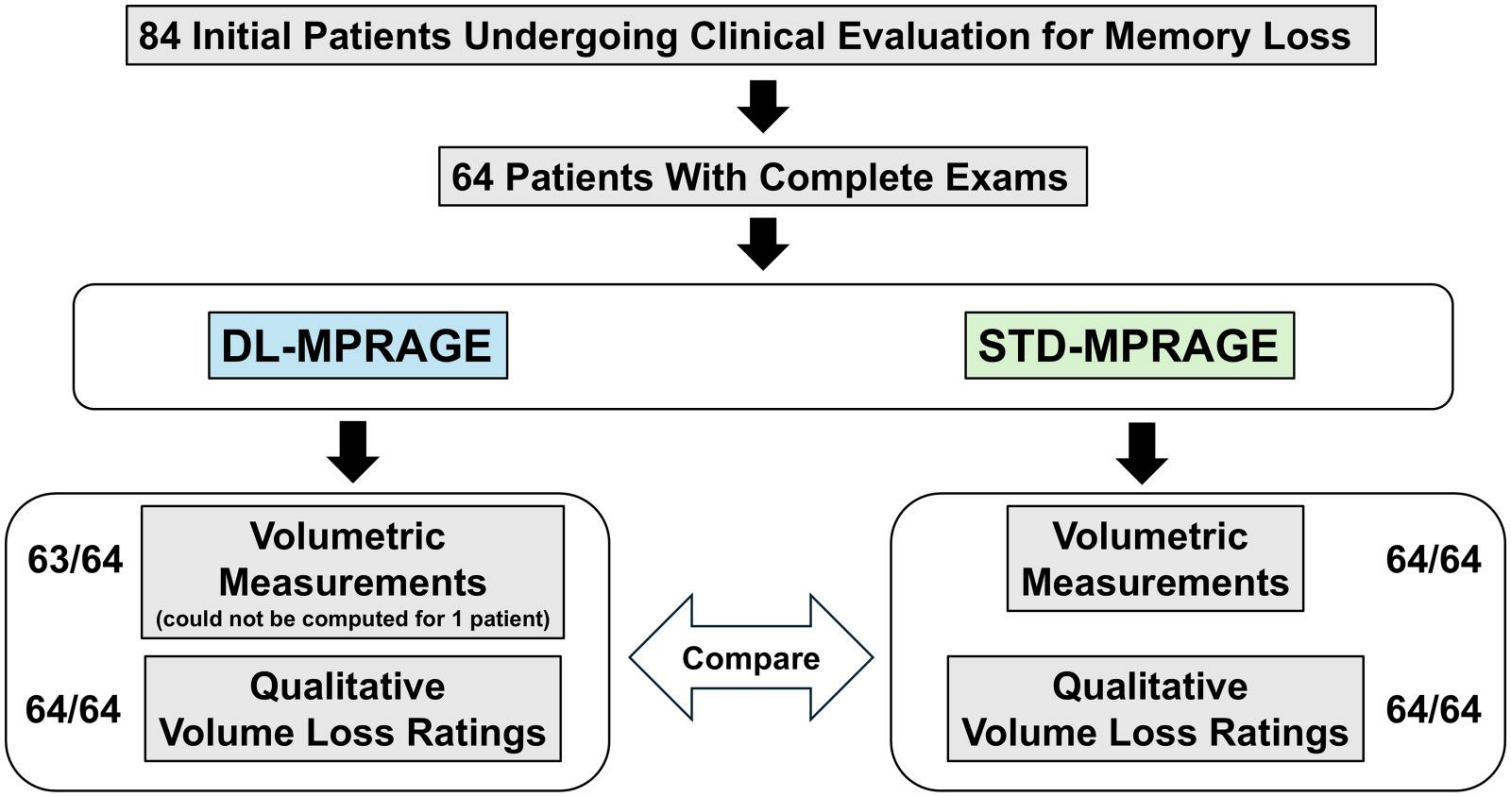
Prospective study design comparing DL-MPRAGE to STD- MPRAGE. Of 84 initially recruited patients, 64 patients had complete examinations including both scans. Volumetric measurements could be obtained for 63 DL-MPRAGE scans and 64 STD-MPRAGE scans. Qualitative evaluations were performed on 64 patients for both DL-MPRAGE and STD-MPRAGE scans. Abbreviations: DL-MPRAGE = deep-learning accelerated MPRAGE; MPRAGE = magnetization-prepared rapid gradient echo; STD-MPRAGE = standard MPRAGE.

This prospective study was approved by the Mass General Brigham institutional review board and is Health Insurance Portability and Accountability Act–compliant. Eighty-four consecutive participants undergoing brain MRI for clinical evaluation of memory loss were scanned between October 2023 and January 2024, among whom 64 obtained complete examinations and were included in this study (Figure 1). Considerations regarding patient consent are detailed as Supplemental Information. The inclusion criterion was referrals for outpatient brain MRI for evaluation of memory loss requiring 3D T1-weighted volumetric imaging. Exclusion criteria were contraindications to MRI, such as claustrophobia and implants, severe motion (as assessed by a senior neuroradiologist with 14 years of experience), and incomplete examinations. For all included patients with complete examinations, we compared differences in volumetric quantification and qualitative assessment obtained with DL-MPRAGE and STD-MPRAGE.

### Image acquisition

All participants underwent an institutional memory loss protocol MRI examination on 3T scanners (MAGNETOM Vida, Siemens Healthineers, Forchheim, Germany), including STD-MPRAGE and DL-MPRAGE sequences. DL-MPRAGE and STD-MPRAGE sequence parameters are reported in Table 1. The acceleration for both DL-MPRAGE and generalized autocalibrating partially parallel acquisition (GRAPPA) was based on parallel imaging, with DL-MPRAGE using controlled aliasing in parallel imaging results in higher acceleration^17^ and STD-MPRAGE using GRAPPA.^18^ More detailed information on the memory loss protocol is provided in Table S1.

**Table 1.** Acquisition parameters for DL-MPRAGE and STD-MPRAGE brain MRI.

Parameter	DL-MPRAGE	**STD-MPRAGE** ^a^
**Resolution (mm^3^)**	0.5 × 0.5 × 1	1 × 1 × 1.1
**Acquisition time (min: sec)**	2:11	5:12
**Imaging plane**	Sagittal	Sagittal
**TR/TE/TI (ms)**	2440/3.98/900	2300/2.98/900
**Field of view (mm^3^)**	256 × 256 × 192	256 × 248 × 193.6
**Matrix**	256 × 256 × 192	256 × 256 × 176
**Interpolation**	Off	Off
**Receiver band width (Hz/pixel)**	360	240
**Acceleration^a^**	2 x 2	2 x 1
**Flip angle (degrees)**	8	9
**Turbo factor**	192	176

^a^DL-MPRAGE results in higher spatial resolution and reduces acquisition time compared to STD-MPRAGE. DL-MRPAGE uses CAIPRINHA for acceleration and STD-MPRAGE uses GRAPPA.

Abbreviations: CAIPIRINHA = controlled aliasing in parallel imaging results in higher acceleration; DL-MPRAGE = deep-learning accelerated MPRAGE; GRAPPA = generalized autocalibrating partially parallel acquisition; MPRAGE = magnetization-prepared rapid gradient echo; STD-MPRAGE = standard MPRAGE; TE = echo time; TI = inversion time; TR = repetition time.

In addition, to qualitatively compare characteristics of STD-MPRAGE and DL-MPRAGE sequences, we performed a scan of the American College of Radiology (ACR) phantom^19^ on the same 3T scanners (MAGNETOM Vida) used for patients.

### DL-MPRAGE sequence

The research application DL-MPRAGE (Figure S1, Table 1) uses a 2-step deep-learning-based image reconstruction that uses an unrolled variational network architecture^16^ for generating images with the acquired resolution, followed by a DL-based superresolution for interpolation to the target resolution.^20^ Methodological details regarding the DL-MPRAGE sequence^21^ can be found in Supplemental Information.

### Quantitative volumetric assessment

Computations of volume and thickness across anatomical regions were performed with FreeSurfer. We estimated volume and thickness from the DL- and STD-MPRAGE images for each patient with the longitudinal FreeSurfer pipeline^22^ in several brain regions (11 for volume and 6 for thickness) and the total brain. The 11 brain regions that were compared for the volume were the frontal, temporal, parietal, occipital lobes, the cingulate gyrus, the insula, the hippocampus, the basal ganglia, the brain stem, the cerebellum, and the cerebral white matter. The 6 brain regions that were compared for the thickness were the frontal, temporal, parietal, and occipital lobes, the cingulate gyrus, and the insula. We quantified differences between the cortical volumes and thicknesses of the two sequences across patients with the absolute symmetrized percent change (ASPC), which is defined for volume comparisons as:

$$\mathrm{ASPC}=100*\frac{\left|\mathrm{Volum}e_{DL}-\mathrm{Volum}e_{\mathrm{STD}}\right|}{0.5*(\mathrm{Volum}e_{DL}+V\mathrm{olum}e_{\mathrm{STD}})}$$

Following prior work,^8^ we generally used the ASPC as a difference measure rather than a standard percentage difference because the ASPC is more robust to the ordering and magnitude of differences in signal.

### Image quality and brain volume loss ratings

Two radiologists, 1 (S.B.) a fellowship-trained neuroradiologist with 2 years of posttraining experience (>50% neuroradiology services) and the other (A.H.) a general radiologist with 14 years of posttraining experience (<50% neuroradiology services), compared DL-MPRAGE and STD-MPRAGE images for all 64 patients by independently rating perceived differences between the 2 sequences in artifacts, sharpness, gray–white matter differentiation, and overall image quality on an integer scale from –2 to +2. Negative ratings indicated that the rater favored STD-MPRAGE and positive ratings indicated that the rater favored DL-MPRAGE. Assigned integer ratings were tabulated in confusion matrices and visualized in a balloon plot—see Supplemental Information for further details.

The 2 blinded radiologists also independently rated levels of cortical volume loss for 6 separate anatomical regions (the anterotemporal, cingulate, frontoinsular, medial-temporal, orbitofrontal, and posterior regions) based on a standardized rating scale,^23^ which assigns integer scores of either 0–3 or 0–4 depending on the anatomical region, with higher numbers correlating to greater volume loss, and mean differences in ratings were computed. See Supplemental Information for further details.

### Interrater agreement

To assess interrater agreement for the quality metrics, we collapsed the 5 possible integer ratings into “Favors STD-MPRAGE,” “No Clear Favorite,” and “Favors DL-MPRAGE” and calculated unweighted Cohen’s kappa values. We also alternatively grouped the ratings into dichotomous “Favors STD-MPRAGE” and “Does Not Favor STD-MPRAGE” categories and constructed 2 × 2 confusion matrices. See Supplemental Information for further details.^24^

### Statistical analysis

Statistical analyses were performed using R version 4.3.3. Differences between the volumes and thicknesses of DL-MPRAGE and STD-MPRAGE were tested for normal distributions based on the Shapiro-Wilk test, with a threshold for departure from normality of *P < *.0026. Statistical comparisons of volumes or thicknesses of anatomical regions were performed using paired Student’s *t*-tests for those anatomical regions whose differences were normally distributed, and with paired Wilcoxon signed-rank tests for those whose differences were not normally distributed. Thresholds for significant differences were *P < *.004 for volume and *P < *.007 for thickness. Comparisons of qualitative ratings of image quality metrics used a significance threshold of *P < *.01. See Supplemental Information for how significance thresholds were determined.

We performed Bland-Altman analysis to compare volumetric measurements for each anatomical region and the total brain and to compare percent differences in volumetric measurements for the total brain.

Comparisons of image quality and volume loss ratings of DL-MPRAGE relative to STD-MPRAGE were performed using 1-sample Wilcoxon signed-rank tests; analyses of the dichotomized rater preferences were performed with the McNemar test. For noninferiority analysis of image quality ratings, we used a noninferiority difference threshold of 0.25,^25^ for which the minimum required sample size at an α = 0.025 and power of 0.90 would be 63 patients.^26^

## Results

### Study population

A total of 64 consecutive participants (29 female, 64.2 ± 16 years old) undergoing brain MRI for clinical evaluation of memory loss were included (Figure 1). Twenty participants were excluded because of incomplete scans that lacked either DL-MPRAGE or STD-MPRAGE sequences. No patients were excluded for severe motion. The clinical indications for brain MRI were clinical concern for memory loss/dementia or evaluation of memory loss symptoms secondary to traumatic brain injury (Table 2); all patients in the study were scanned with the same MRI protocol. The DL-MPRAGE images for 1 patient could not complete FreeSurfer volumetric calculations and were excluded from quantitative analysis.

**Table 2.** Summary characteristics of the study population undergoing brain MRI for memory loss.

**Number of subjects**	64^a^
**Mean age (years ± SD)**	62.4 ± 16
**Sex (female: male)**	29:35
**Clinical indication for MRI**	N (%)
Memory loss Traumatic brain injury	53 (83%) 11 (17%)

a 64 patients were included for qualitative analysis, and 63 patients were included for quantitative analysis. In 1 patient, quantitative data could not be calculated.

### Time savings of DL-MPRAGE compared to STD-MPRAGE

**Figure 2.**
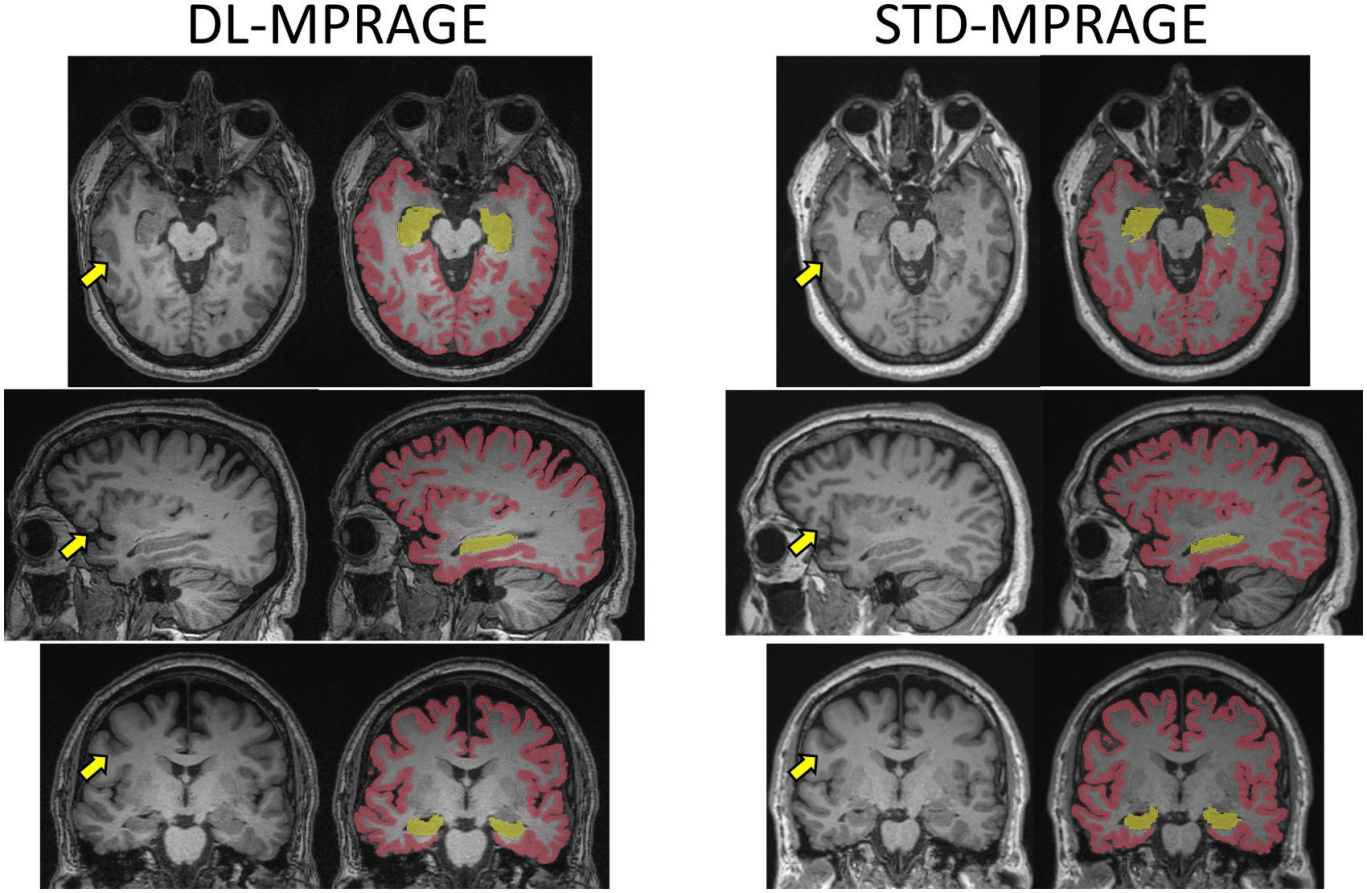
Qualitative comparison between DL-MPRAGE and STD-MPRAGE demonstrating axial, sagittal, and coronal slices and FreeSurfer segmentations of a single patient. DL-MPRAGE demonstrates qualitatively similar delineation of sulci and gyri (examples highlighted by yellow arrows) based on automated FreeSurfer segmentations. Segmentations of the cerebral cortex (red) and hippocampus (yellow) are specifically highlighted. Abbreviations: DL-MPRAGE = deep-learning accelerated MPRAGE; MPRAGE = magnetization-prepared rapid gradient echo; STD-MPRAGE = standard MPRAGE.

The acquisition time of DL-MPRAGE was 40% that of STD-MPRAGE (2 min, 11 sec vs. 5 min, 21 sec; Table 1). For our institutional memory loss protocol, this translates to a protocol using DL-MPRAGE taking 14 min and 11 seconds, versus one using STD-MPRAGE taking 16 min and 11 seconds, for a time saving of 15% (Table S1). DL-MPRAGE also achieved a spatial resolution of 0.5 mm in the axial plane due to the superresolution algorithm, improving on the 1.0-mm resolution of STD-MPRAGE. Visually, DL-MPRAGE scans demonstrated overall similar delineation of sulci and gyri throughout the brain compared to STD-MPRAGE (Figure 2). Automated FreeSurfer segmentations of the inner and outer cortical surfaces were also qualitatively similar (Figure 2), as well as of deeper anatomic structures such as the brain stem (Figure S2).

### Quantitative volumetric comparison of DL-MPRAGE and STD-MPRAGE

**Figure 3.**
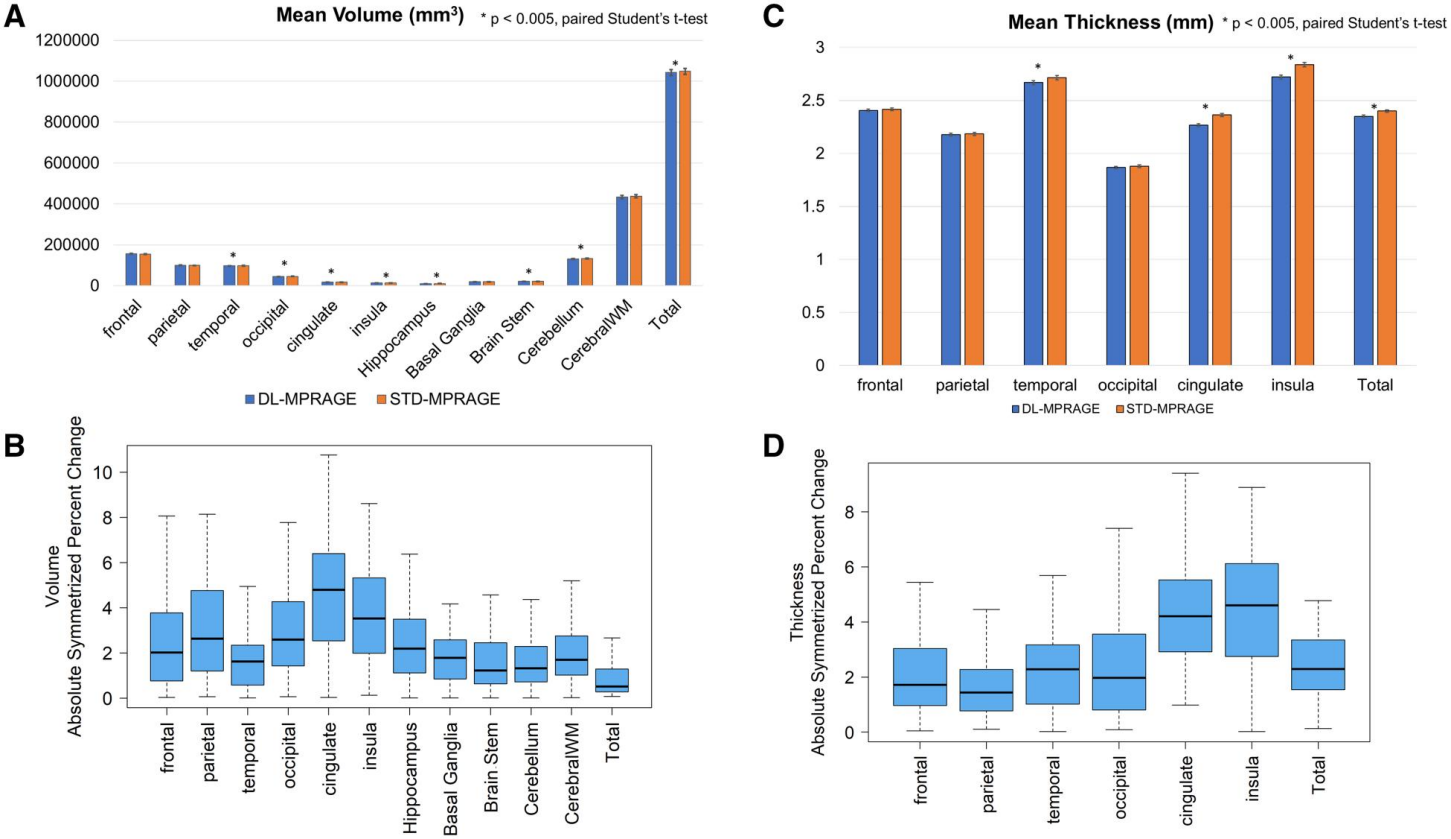
(A) Mean volumes across anatomical regions for DL-MPRAGE and STD-MPRAGE show that although some differences are statistically significant, they are small in relative magnitude. (B) Boxplot of volume absolute symmetrized percent change (ASPC) comparing DL-MPRAGE with STD-MPRAGE. Median values (thick black bars) and interquartile ranges (IQRs; blue boxes) are plotted. Boxplot whisker tips extend to the most extreme data points within ±1.5 × IQR. (C) Mean thicknesses across anatomical regions for DL-MPRAGE and STD-MPRAGE show that although some differences are statistically significant, they are small in relative magnitude. (D) Boxplot of thickness ASPC comparing DL-MPRAGE with STD-MPRAGE. Median values (thick black bars) and IQRs (blue boxes) are plotted. Boxplot whisker tips extend to the most extreme data points within ±1.5 × IQR. Abbreviations: ASPC = absolute symmetrized percent change; DL-MPRAGE = deep-learning accelerated MPRAGE; MPRAGE = magnetization-prepared rapid gradient echo; STD-MPRAGE = standard MPRAGE.

Volumetric calculations of cortical volume and thickness were completed for 63 patients with DL-MPRAGE scans. Volumetric calculations for 1 patient could not be completed because FreeSurfer was not able to process the DL-MPRAGE images because of the field of view parameter being too large. Overall, the differences between the calculated volumes and thicknesses for DL-MPRAGE and STD-MPRAGE were normally distributed for most anatomical regions (Shapiro-Wilk *P* > .0026). Departures from normality occurred for the volume differences of the parietal lobe, cingulate gyrus, and basal ganglia, and for the thickness differences of the insula (Shapiro-Wilk *P < *.0026 for these distributions). Calculated cortical volumes were comparable between DL-MPRAGE and STD-MPRAGE, but with statistically significant (*P < *.004) differences in certain anatomical regions (Figure 3A; Table 3): the temporal (96 832 ± 11 435 vs. 97 977 ± 11 882 mm^3^) and occipital (44 876 ± 6617 vs. 45 669 ± 6992 mm^3^) lobes, the cingulate gyrus (17 226 ± 2011 vs. 17 924 ± 2185 mm^3^), the insula (12 800 ± 1419 vs. 13 216 ± 1556 mm^3^), hippocampus (10 349 ± 1826 vs. 10 529 ± 1793 mm^3^), and brain stem (21 288 ± 2962 vs. 21 358 ± 2810 mm^3^), and the total brain (1 042 504 ± 114 523 vs. 1 047 889 ± 112 097 mm^3^). Nevertheless, ASPC values quantifying the differences were small, with medians averaging 2.31% with a maximum of 4.79 ± 3.89% for the cingulate gyrus (Figure 3B). Similarly, the cortical thicknesses demonstrated small statistically significant (*P < *.007) differences in the temporal lobe (2.67 ± 0.15 vs. 2.72 ± 0.16 mm), cingulate gyrus (2.27 ± 0.11 vs. 2.36 ± 0.12 mm), insula (2.72 ± 0.14 vs. 2.84 ± 0.17 mm), and total brain (2.35 ± 0.09 vs. 2.40 ± 0.10 mm) (Figure 3C; Table 3). The cingulate gyrus (4.21 ± 4.71%) and insula (4.60 ± 3.40%) revealed the largest ASPC though they still remained less than 5% (Figure 3D).

**Table 3.** Quantitative cerebral volume calculations with DL-MPRAGE and STD-MPRAGE.

Volume (mm^3^)				
Anatomical Region	DL-MPRAGE	STD-MPRAGE	ASPC	*P* value
	Mean ± SD	Mean ± SD	Median ± IQR	
Frontal	155 352 ± 15 690	153 722 ± 16 177	2.02 ± 3.06	.0217
Parietal	99 549 ± 12 613	98 676 ± 12 075	2.63 ± 3.72	.006**
Temporal	96 832 ± 11 435	97 977 ± 11 882	1.61 ± 1.80	<.004*
Occipital	44 876 ± 6617	45 669 ± 6992	2.59 ± 2.86	<.004*
Cingulate	17 226 ± 2011	17 924 ± 2185	4.79 ± 3.89	<.004*^,^**
Insula	12 800 ± 1419	13 216 ± 1556	3.52 ± 3.35	<.004*
Hippocampus	10 349 ± 1826	10 529 ± 1793	2.19 ± 2.39	<.004*
Basal ganglia	19 422 ± 3118	19 446 ± 2876	1.78 ± 1.77	.755**
Brain stem	21 288 ± 2962	21 358 ± 2810	1.23 ± 1.90	.303
Cerebellum	130 989 ± 12 799	132 451 ± 12 884	1.33 ± 1.71	<.004*
Cerebral white matter	433 821 ± 63355	436 922 ± 59 509	1.70 ± 1.77	.0157
Total	1 042 504 ± 114 523	1 047 889 ± 112 097	0.52 ± 1.04	<.004*
Cortical Thickness (mm)				
Frontal	2.41 ± 0.09	2.42 ± 0.10	1.72 ± 2.12	.121
Parietal	2.18 ± 0.10	2.18 ± 0.12	1.44 ± 1.59	.235
Temporal	2.67 ± 0.15	2.72 ± 0.16	2.28 ± 2.24	<.007*
Occipital	1.87 ± 0.09	1.88 ± 0.11	1.97 ± 2.84	.0796
Cingulate	2.27 ± 0.11	2.36 ± 0.12	4.21 ± 2.71	<.007*
Insula	2.72 ± 0.14	2.84 ± 0.17	4.60 ± 3.40	<.007*^,^**
Total	2.35 ± 0.09	2.40 ± 0.10	2.29 ± 1.83	<.007*

One patient was excluded because of the inability to complete FreeSurfer volumetric calculations. Statistically significant *P* values (<.004 for volume and <.007 for thickness) are indicated with an asterisk (*).

** Paired Wilcoxon signed-rank test *P*-value.

Abbreviations: ASPC = absolute symmetrized percent change; DL-MPRAGE = deep-learning accelerated MPRAGE; MPRAGE = magnetization-prepared rapid gradient echo; SD = standard deviation; STD-MPRAGE = standard MPRAGE.

**Figure 4.**
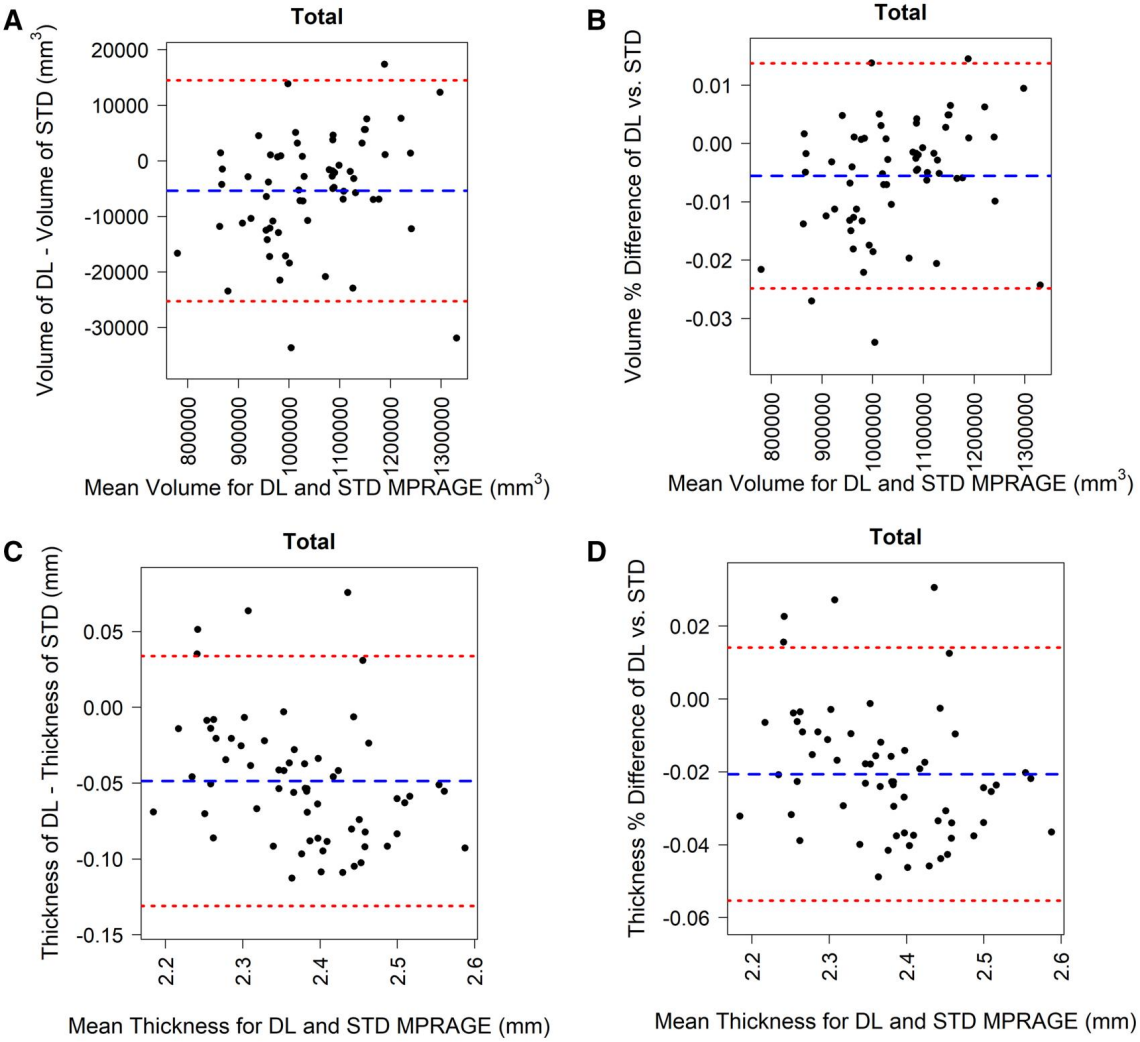
Bland-Altman analysis of 64 patients comparing raw (A) and percent (B) differences in volume, and raw (C) and percent (D) differences in thickness of the total brain as a function of the mean volume and thickness between DL-MPRAGE and STD-MPRAGE. The blue dashed lines in each plot correspond to the mean of the y-axis, and the red dashed lines correspond to the mean ± 1.96 × SD of the differences. Almost all points lie between the 95% limits of agreement for both volume and thickness. Abbreviations: DL-MPRAGE = deep-learning accelerated MPRAGE; MPRAGE = magnetization-prepared rapid gradient echo; STD-MPRAGE = standard MPRAGE.

To illustrate the similarity between morphometric measurements for DL-MPRAGE and STD-MPRAGE, we performed Bland-Altman analysis for each anatomical region and the total brain (Figure 4). Distributions of both raw (Figure 4A) and percent (Figure 4B) differences in total brain volume and raw (Figure 4C) and percent (Figure 4D) differences in mean total cortical thickness suggested general agreement between DL-MPRAGE and STD-MPRAGE. This pattern was observed for individual anatomical regions as well, both for volumes (Figure S3) and thicknesses (Figure S4).

### Qualitative comparison of DL-MPRAGE and STD-MPRAGE

**Figure 5.**
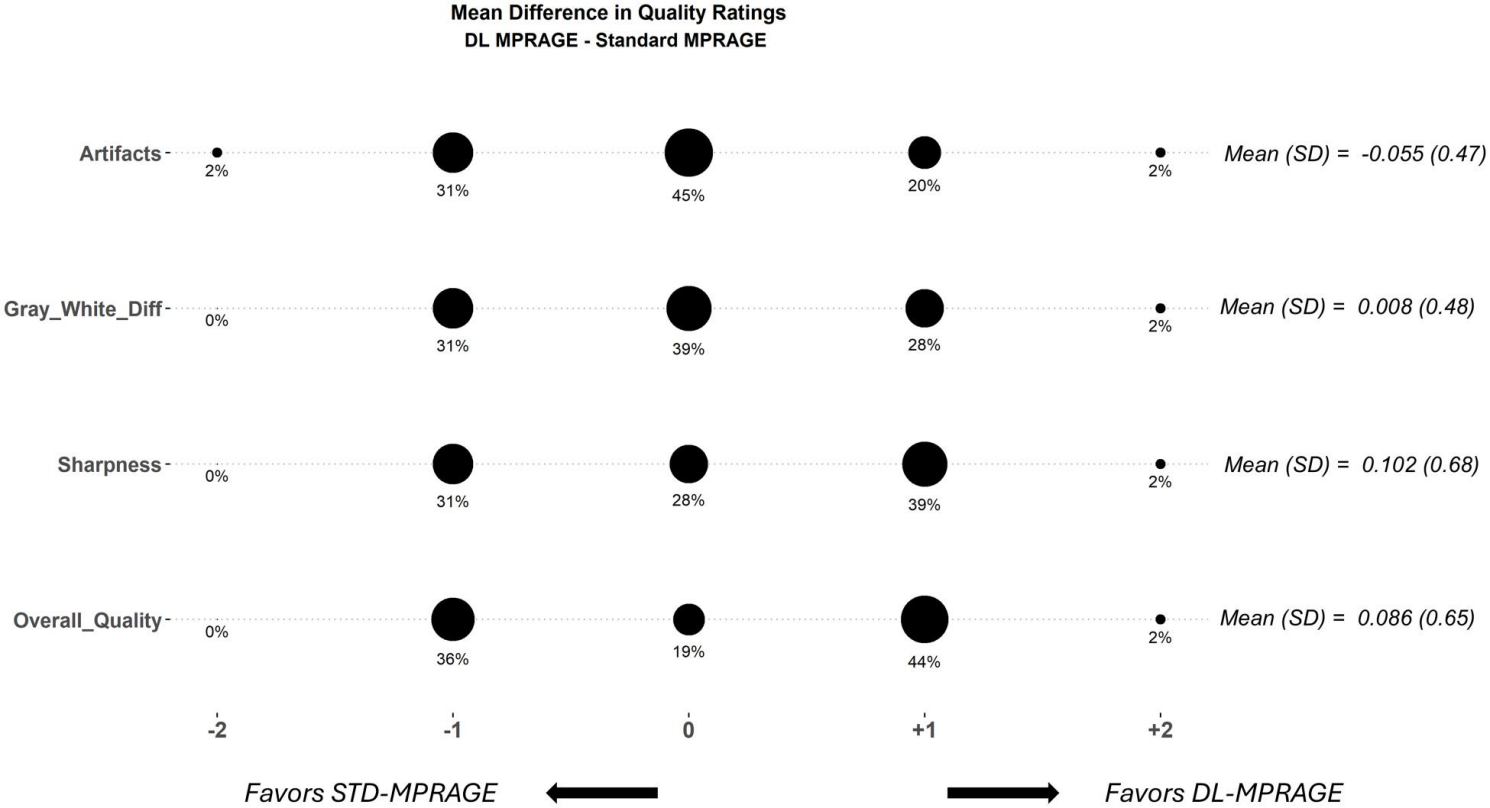
Balloon plot of mean qualitative image quality rating differences between DL- and STD-MPRAGE in 64 patients, averaged for 2 raters and rounded to the nearest integer, for 4 categories. The sizes of the balloons are proportional to the percentage of cases that were assigned a particular mean rating. For each qualitative category, positive ratings indicate DL-MPRAGE is favored, and negative ratings indicate STD-MPRAGE is favored. Overall, DL-MPRAGE was noninferior to STD-MPRAGE in all categories (*P < *.01 for all categories). Abbreviations: DL-MPRAGE = deep-learning-accelerated MPRAGE; Gray-White-Diff = Gray-White Differentiation; STD-MPRAGE = standard MPRAGE; SD = standard deviation.

**Figure 6.**
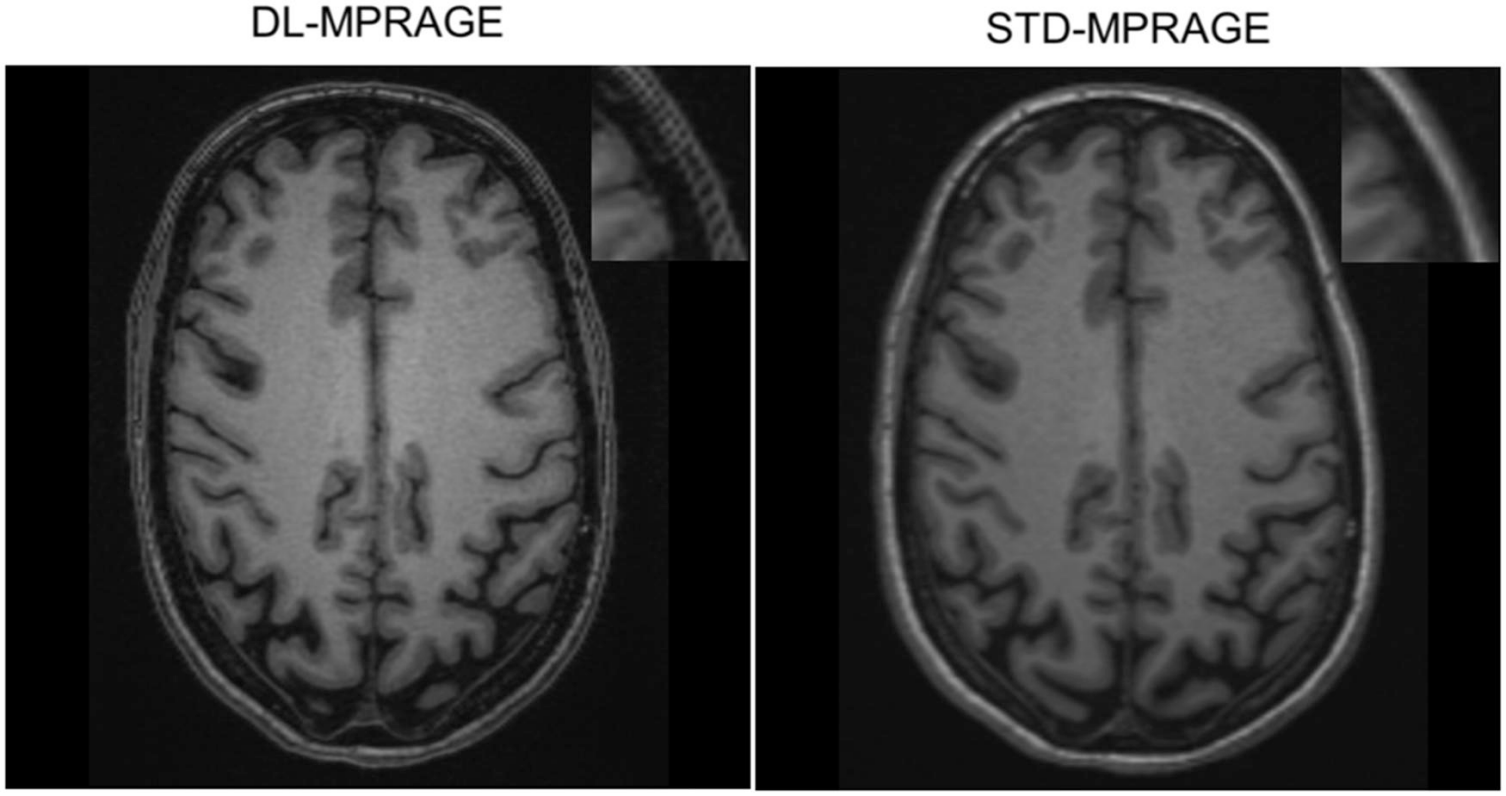
Example of alternating bands of hyperintensity/hypointensity (Gibbs ringing artifact) adjacent to the calvarium in the scalp for one subject observed with the DL-MPRAGE sequence (magnified in the top-right inlet). Abbreviation: DL-MPRAGE = deep-learning accelerated MPRAGE; MPRAGE = magnetization-prepared rapid gradient echo.

Comparisons of the 2 blinded radiologists’ subjective image quality ratings between DL-MPRAGE and STD-MPRAGE demonstrated that DL-MPRAGE was noninferior to STD-MPRAGE in perceived image sharpness, artifacts, gray–white differentiation, and overall quality (*P < *.001 for all) (Figure 5). DL-MPRAGE demonstrated slight advantages in sharpness (*P* = .260) and overall image quality (*P* = .308) but also a slightly higher artifact rating (*P* = .319), although these differences could not be shown as statistically significant; it also showed essentially no difference in gray–white differentiation (*P* = .982). As an example, the raters found that DL-MPRAGE images tended to exhibit areas adjacent to the calvarium with alternating bands of hyper-/hypointensity (Gibbs ringing artifact; Figure 6). In addition, we performed a test of DL-MPRAGE and STD-MPRAGE on the ACR phantom,^19^ qualitatively demonstrating that although DL-MPRAGE and STD-MPRAGE are similar in overall image quality, DL-MPRAGE is prone to ringing artifact, independent of motion (Figure S5).

There were no significant differences in the qualitative cortical volume loss ratings between the two blinded radiologists, with complete agreement in the ratings of the posterior, medial temporal, and anterotemporal regions. The mean differences in the ratings of the remaining regions (orbitofrontal, frontoinsular, and cingulate) were also nearly zero (Table 4). No differences in volume loss ratings were statistically significantly different from zero.

**Table 4.** Qualitative volume loss ratings between DL-MPRAGE and STD-MPRAGE.

Anatomical Region	Mean Volume Loss Rating Difference Value (Standard Deviation)
Anterotemporal	0 (0)
Cingulate	0.016 (0.12)
Frontoinsular	0.047 (0.21)
Medial-Temporal	0 (0)
Orbitofrontal	0.031 (0.25)
Posterior	0 (0)

The mean differences in volume loss ratings were not statistically significantly different from zero in any anatomical region (Wilcoxon rank-sum test *P* > .05).

Abbreviations: DL-MPRAGE = deep-learning accelerated MPRAGE; MPRAGE = magnetization-prepared rapid gradient echo; STD-MPRAGE = standard MPRAGE.

### Interrater agreement

Interrater analysis was performed to assess agreement between the 2 raters in the assessment of image quality metrics. Unweighted Cohen’s kappa values suggested near-perfect agreement in ratings for overall quality (κ = 0.88) and gray–white differentiation (κ = 0.91), and substantial agreement in ratings for sharpness (κ = 0.70) and artifacts (κ = 0.77) (Table S2). Confusion matrices from which the interrater agreement was derived are provided in Figure S6.

Analysis of the interrater agreement considering the ratings as dichotomous variables (Figure S7) suggests statistically significant differences (McNemar *P < *.01) between rater classifications for Overall Quality, Gray–White Differentiation, and Artifacts, and no significant difference for Sharpness (McNemar *P* = .028), although the *P* value for Sharpness approaches the significance threshold.

## Discussion

This study of DL-MPRAGE represents a clinical evaluation of physics-based DL-accelerated imaging and super-resolution. The use of brain MRIs for the evaluation of memory loss including T1-weighted MPRAGE sequences is expected to increase as the arsenal of neurotherapeutics for Alzheimer’s disease grows.^27^ The scan time of DL-MPRAGE is 40% compared to STD-MPRAGE (translating to a reduction in total scan time by 15%) while maintaining noninferior image quality. Reduced scan times enhance scheduling efficiency, facilitating the imaging of patients requiring serial examinations, such as those receiving antiamyloid therapy, in whom timely acquisition is essential for informed clinical management and optimal care delivery.

The key takeaways from our work are as follows: (1) Our study tests a DL-based method that directly takes k-space data as an input, rather than working entirely in the image domain, on a clinical cohort, which is advantageous given few studies demonstrate the deployment of DL-enhanced neuroimaging advances in real-world clinical environments.^28^ (2) In a subset of anatomical regions, DL-MPRAGE produced slightly lower volume/thickness estimates compared to STD-MPRAGE, consistent with other prior studies on AI-enabled superresolution T1-weighted MPRAGE imaging, which may be due to STD-MPRAGE’s reduction of partial volume effects with cerebrospinal fluid and/or white matter in adjacent voxels.^15^^,^^29^ (3) Nevertheless, Bland-Altman analysis and APSC values support general interchangeability between morphometric calculations obtained from DL-MPRAGE and STD-MPRAGE across anatomical regions; furthermore, the median ASPC value of 2.8% that we observed for differences in volume measurements matches prior findings on the limits of reproducibility of FreeSurfer-based volumetric measurements in MPRAGE sequences.^8^ (4) Finally, qualitative assessment suggests that DL-MPRAGE is noninferior to STD-MPRAGE in image quality and equivalent for assessing regional cortical volume loss.

Studies in different clinical settings/populations, such as for parenchymal lesion detection, may reveal more appreciable differences that take advantage of the submillimeter resolution of DL-MPRAGE. For example, 1 prior study examined a DL-based accelerated acquisition and superresolution method on a dataset mostly consisting of brain tumor posttreatment follow-up scans^25^ and found that the DL-enhanced 3D T1-weighted precontrast sequences were rated as superior to corresponding standard-of-care 3D T1-weighted precontrast sequences in overall quality. However, the magnitude of preference was small (mean rating of 4.1 ± 0.7 vs. 4.0 ± 0.7 on a 5-point integer scale averaged over 4 raters), and the difference in mean ratings of 0.1 is comparable to the difference of 0.086 we observed for overall quality (Figure 5). Notably, that study was based on data acquired on 1.5T scanners. All patients in our study were scanned at 3T, which may have increased the baseline signal-to-noise ratio of the scans such that the increased resolution introduced by DL-MPRAGE was qualitatively imperceptible.

One of the earliest studies to implement DL-enhanced brain MRI by Bash et al.^30^ compared standard-of-care 3D T1-weighted sequences to accelerated acquisitions that used k-space undersampling and a reduced imaging matrix (FAST), as well as FAST scans to which a commercial DICOM-based DL image denoising and sharpness-enhancing tool was applied (FAST-DL). FAST-DL scans demonstrated superior subjective quality ratings over standard-of-care scans, with a mean preference for FAST-DL of 0.5 points on a 5-point integer scale, and almost no statistically significant differences in morphometric measures. Although the study by Bash et al. also examined a population of patients being evaluated for memory loss, the results of our study differ in that subjective quality ratings were not statistically different between DL-enhanced and standard-of-care scans. One possible explanation for the difference in findings to a statistical variation introduced by the relatively small sample sizes in both studies (40 in Bash et al, 64 in our work). Another possible explanation is that the FAST-DL scans introduced fewer artifacts and had a stronger denoising effect than DL-MPRAGE. The use of unfiltered k-space data by the unrolled variational network in DL-MPRAGE may have resulted in the introduction of additional artifacts such as Gibbs ringing (Figure 6) that image domain–based deep learning acceleration methods do not produce. The superresolution algorithm used in DL-MPRAGE may represent an additional potential source of noise and artifacts, and may represent an area for potential further optimization, both to correct for the intrinsic introduction of artifacts and to correct for the artifacts introduced by direct manipulation of k-space.

A limitation of the current work is that it is a single-center study and hence requires confirmation in other settings to establish generalizability. Second, although our sample size is larger than other similar clinical validation studies of DL-accelerated brain MRI sequences^25^^,^^30^^,^^31^ to date, a larger data set would maintain high statistical power at lower significance thresholds that could account for more potential sources of statistical variation and could also potentially detect differences in volumetrics or qualitative ratings that were not seen in our study. Third, we only examined volumetrics using FreeSurfer. Small systematic differences in volumetric values exist between FreeSurfer and other methods,^32^^,^^33^ with, for example, total brain volumes being reported as 6.5% larger on average in NeuroQuant compared to FreeSurfer.^32^ The statistically significant differences between DL- and STD-MPRAGE volumetrics we observed were small, but on the order of reported variation between methods of volumetric calculation.

Testing on an external dataset would be the next step toward demonstrating the generalizability of our findings. See Supplemental Information for additional discussion regarding this point in the context of our study.^31^^,^^34^^,^^35^

Although the DL-MPRAGE sequence evaluated in this study is a works-in-progress package specific to Siemens scanners, similar DL-based reconstruction methods, including unrolled variational networks, are under active development by multiple vendors. Although differences in implementation may slightly influence image characteristics, the general approach is expected to become more widely available across platforms and testable. Our results demonstrate that DL-MPRAGE provides essentially equivalent quantitative and qualitative metrics to current standard-of-care imaging while achieving a more than 2-fold reduction in acquisition time. We have tested DL-MPRAGE in our routine clinical protocol for evaluating memory loss, and future work may focus on further clinically validating DL-MPRAGE in a larger, multicenter dataset. In addition, DL-based image acquisition and reconstruction may be compared to MPRAGE sequences obtained with other acceleration strategies, such as Wave-CAIPI MPRAGE.^8^

## Supplementary Material

umaf022_Supplementary_Data

## Data Availability

Data generated or analyzed during the study are available from the corresponding author by request.
