# Supplementary material for: Deep-learning-accelerated T1-MPRAGE MRI for quantification and visual grading of cerebral volume in memory loss patients
Source: Radiol Adv. 2025 Jun 2;2(4):umaf022. doi: 10.1093/radadv/umaf022 (PMC12255235; doi:10.1093/radadv/umaf022)
Supplement: umaf022_Supplementary_Data [file umaf022_Supplementary_Data.zip › DL_STD_MPRAGE_supplemental_R4_clean.docx]

**Supplemental Information**

**Supplemental Methods**

**Patient Consent for Study.** Given that the additional scan time from the DL-MPRAGE sequence was less than 3 min per scan, the institutional review board waived the need for written informed consent. An information sheet presenting a detailed description of the research study was provided to the study participants prior to the scan and given the option to decline participation. All participants were outpatients who were able to read, comprehend, and provide verbal understanding of the study.

**DL-MPRAGE Sequence.**  DL-MPRAGE is currently a Works-In-Progress (WIP) research application provided by Siemens Healthineers, available for MAGNETOM Vida scanners (software platforms VA50/51/60/61). The sequence is not currently cleared by the United States Food and Drug Administration.

The employed research application was previously used for dynamic liver imaging [1]. In the first step the image reconstruction receives acquired k-space data as well as coil sensitivity maps as input and then alternates between a parallel imaging step and a neural network-based image enhancement for a total of 6 iterations. The step sizes and the model parameters of the neural networks were considered as trainable and determined through supervised training using about 5000 training pairs derived from about 500 fully sampled 3D k-space datasets obtained from volunteers using 1.5T and 3T scanners (MAGNETOM scanners, Siemens Healthineers, Forchheim, Germany) in multiple body regions. The parameters were determined with a dedicated GPU server using a PyTorch implementation of the unrolled network architecture and then exported for prospective use in the scanner’s integrated reconstruction pipeline. The inference time for an MPRAGE dataset on the scanner was about 15 sec.

The second step employed a deep learning-based superresolution algorithm that interpolates by a factor of 2 in all spatial directions. The algorithm was trained using about 500 high-resolution, complex-valued images that were down-sampled to generate the input for supervised training [2]. The obtained parameters were again exported and integrated into the scanner’s reconstruction pipeline. Not using the GPU on the scanner, the inference time for an MPRAGE dataset on the scanner was approximately 30 seconds.

**Image Quality and Brain Volume Loss Ratings.** The radiologists examined each pair of sequences in tandem and were blinded to the sequence assignments, with sequences randomly labeled as “DCM1” or “DCM2” for each patient. Negative ratings corresponded to favoring the sequence labeled as “DCM1” and positive ratings corresponded to favoring the sequence labeled as “DCM2”. Ratings were derandomized and adjusted by a different unblinded investigator to have positive ratings correspond to favoring DL-MPRAGE and negative ratings correspond to favoring STD-MPRAGE. The numerical rating values were averaged between the two raters and rounded to the nearest integer for visualization purposes in a balloon plot and left unrounded for statistical analysis. Assigned integer ratings for the two raters were tabulated in confusion matrices for each of the image quality ratings. Ratings of -2 represented that the rater “strongly favored STD-MPRAGE”, -1 that the rater “weakly favored STD-MPRAGE”, 0 that the rater had “no favorite”, 1 that the rater “weakly favored DL-MPRAGE”, and 2 that the rater “strongly favored DL-MPRAGE”.

Qualitative brain volume loss ratings were initially assigned separately for right and left anatomical regions. Mean differences in ratings between DL- and STD-MPRAGE for each anatomical region were computed and right and left components averaged. The volume loss rating values were left unrounded for statistical analyses.

**Interrater Agreement.** We collapsed the five possible ratings (-2, -1, 0, 1, 2) into three categories: -2, -1 were marked as “Favors STD-MPRAGE", 0 was marked as “No Clear Favorite”, and 1, 2 were marked as “Favors DL-MPRAGE". Instances where one rater marked an original score of 1 or –1 and the other marked an original score of 0 were treated as being in the “No Clear Favorite” category. Interrater agreement using the three collapsed categories for the four quality ratings was calculated using unweighted Cohen’s kappa values, and 95% confidence intervals were calculated as in Fleiss, Cohen, et al [3].

Additionally, we performed an alternative analysis of interrater agreement by grouping the ratings into “Favors STD-MPRAGE” and “Does Not Favor STD-MPRAGE”. The numbers of cases assigned to each of the two categories by the two raters were tabulated in 2x2 confusion matrices for each of the four image quality metrics.

**Significance Thresholds in Statistical Analysis.** Regarding tests for normality, applying Bonferroni correction to the 19 distributions tested (12 for volume differences, 7 for thickness differences), the threshold for significance from departure from normality was set to p < 0.0026.

Significance thresholds were adjusted by Bonferroni correction of 12 statistical comparisons for the volumes (the 11 anatomical regions and the total brain) and 7 comparisons for the thicknesses. For an α = 0.05, this yields statistical significance thresholds of 0.05/12 = 0.004 for the volumes and 0.05/7 = 0.007 for the thicknesses.

For comparisons involving the four image quality metrics, Bonferroni correction of an α = 0.05 suggested a corrected significance threshold of 0.05/4 = 0.013, abbreviated as 0.01.

**Supplemental Figures and Tables**


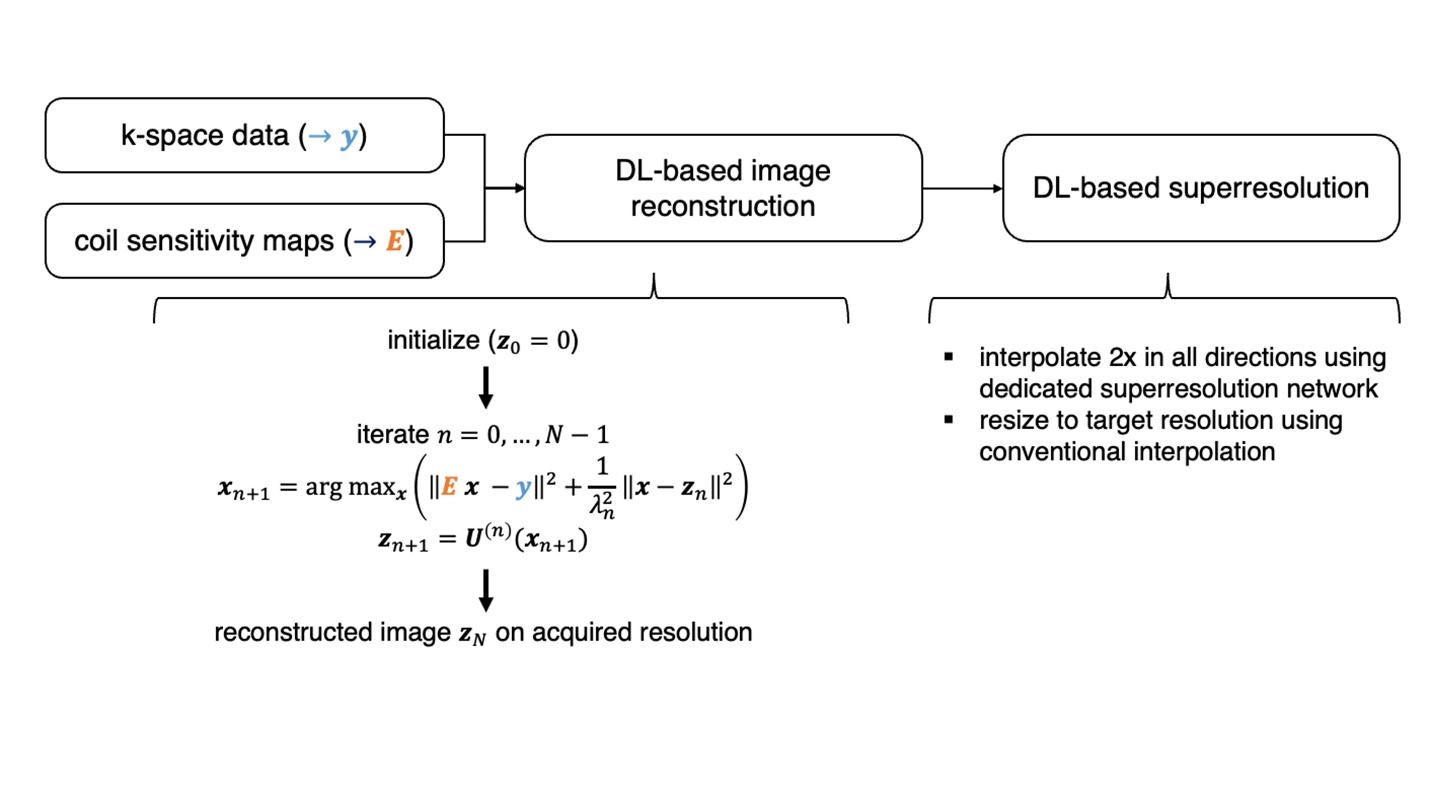


**Figure S1.** Diagram of deep learning-based reconstruction technique applied for DL-MPRAGE in this study. The key strength of unrolled networks lies in their ability to directly incorporate MRI physics into the neural network architecture. The algorithm receives acquired k-space data *y* and coil sensitivity maps *E* as inputs and generates an image *z_N_* on the acquired resolution using an unrolled network that alternates between parallel imaging-based data consistency (*x*) and image enhancement based on neural network *U* for *N* iterations with step size *λ_n_*. The obtained image is further interpolated using a deep learning-based superresolution.


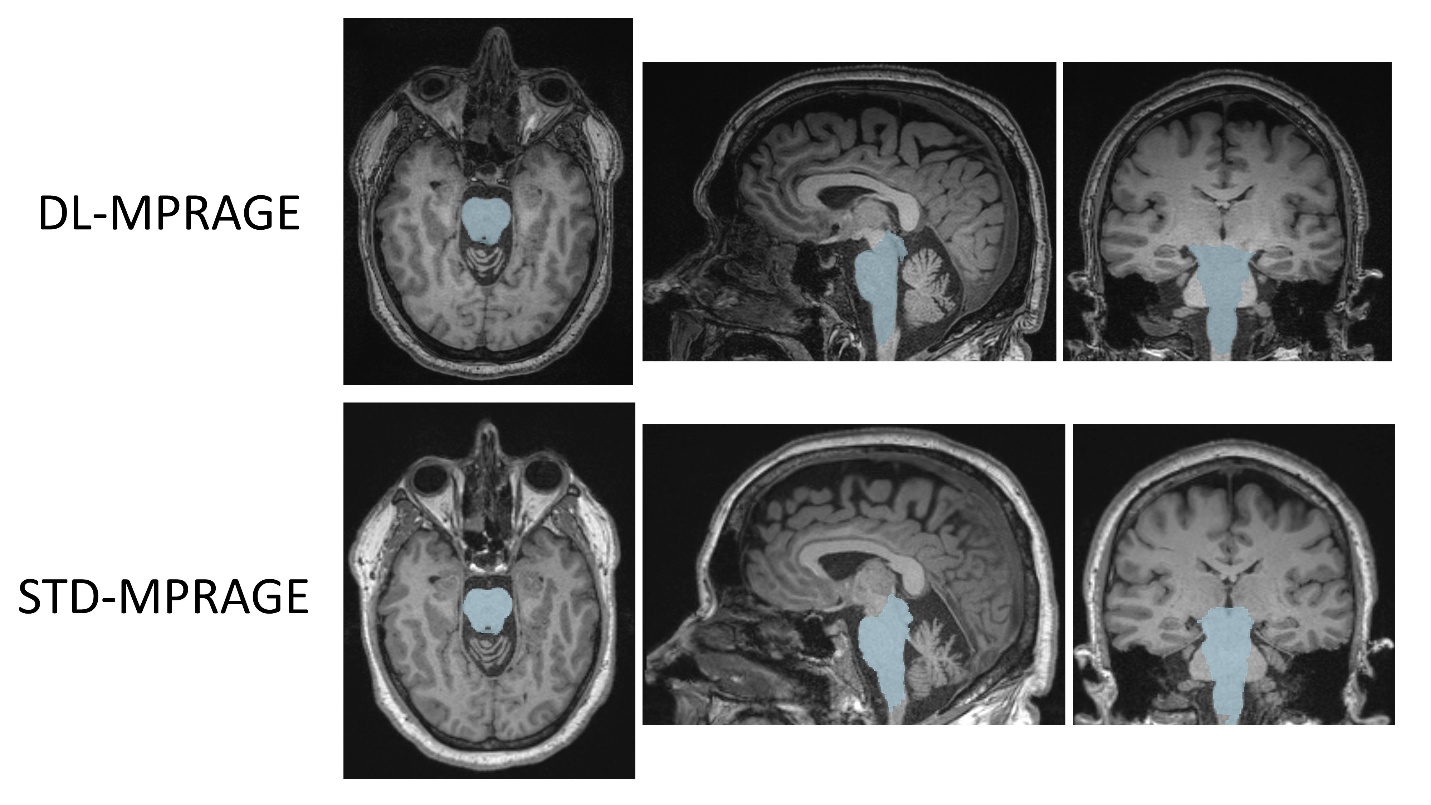


**Figure S2.** Qualitative comparison of brain stem segmentation between DL-MPRAGE and STD-MPRAGE in a single patient. The segmentation of the brain stem is virtually identical between DL-MPRAGE and STD-MPRAGE.


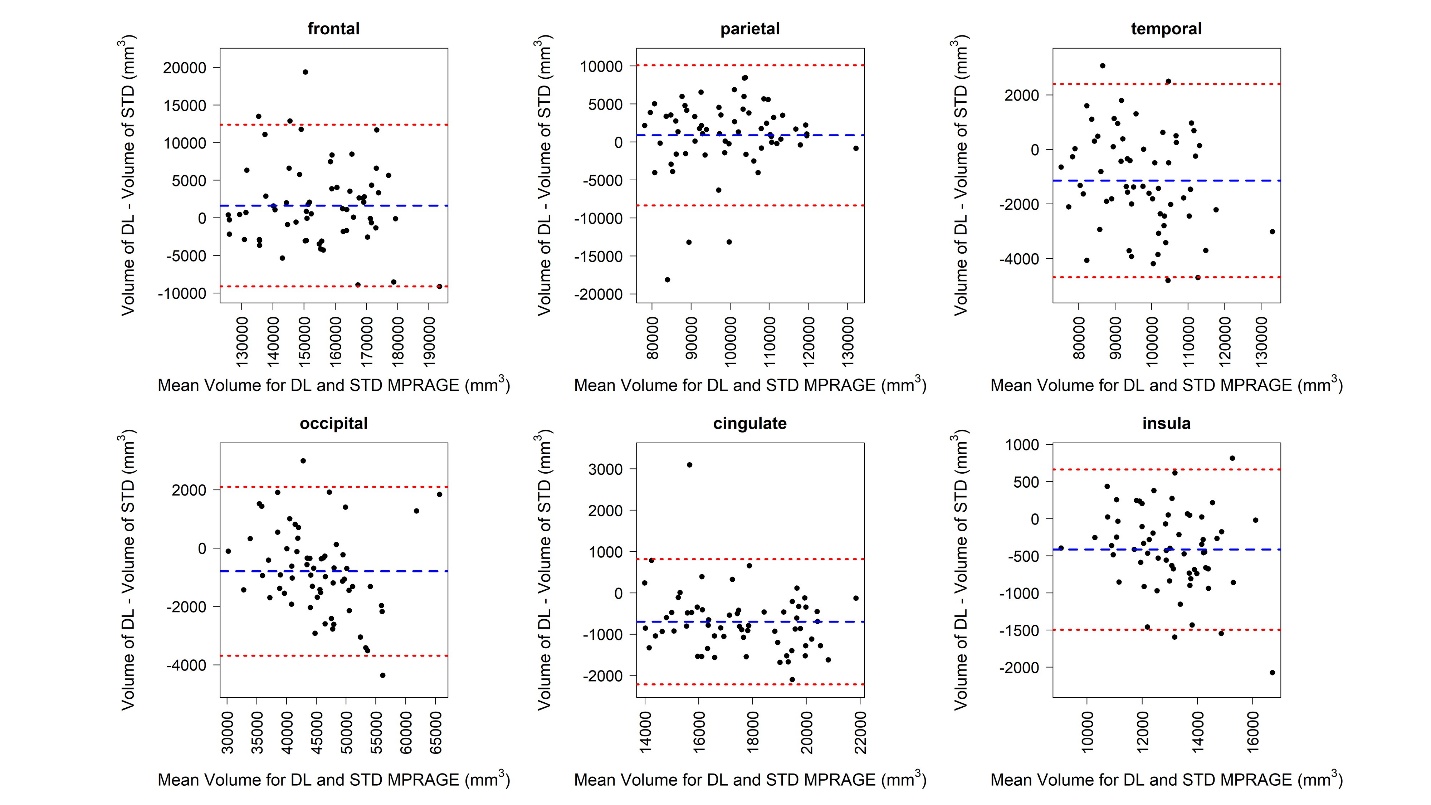

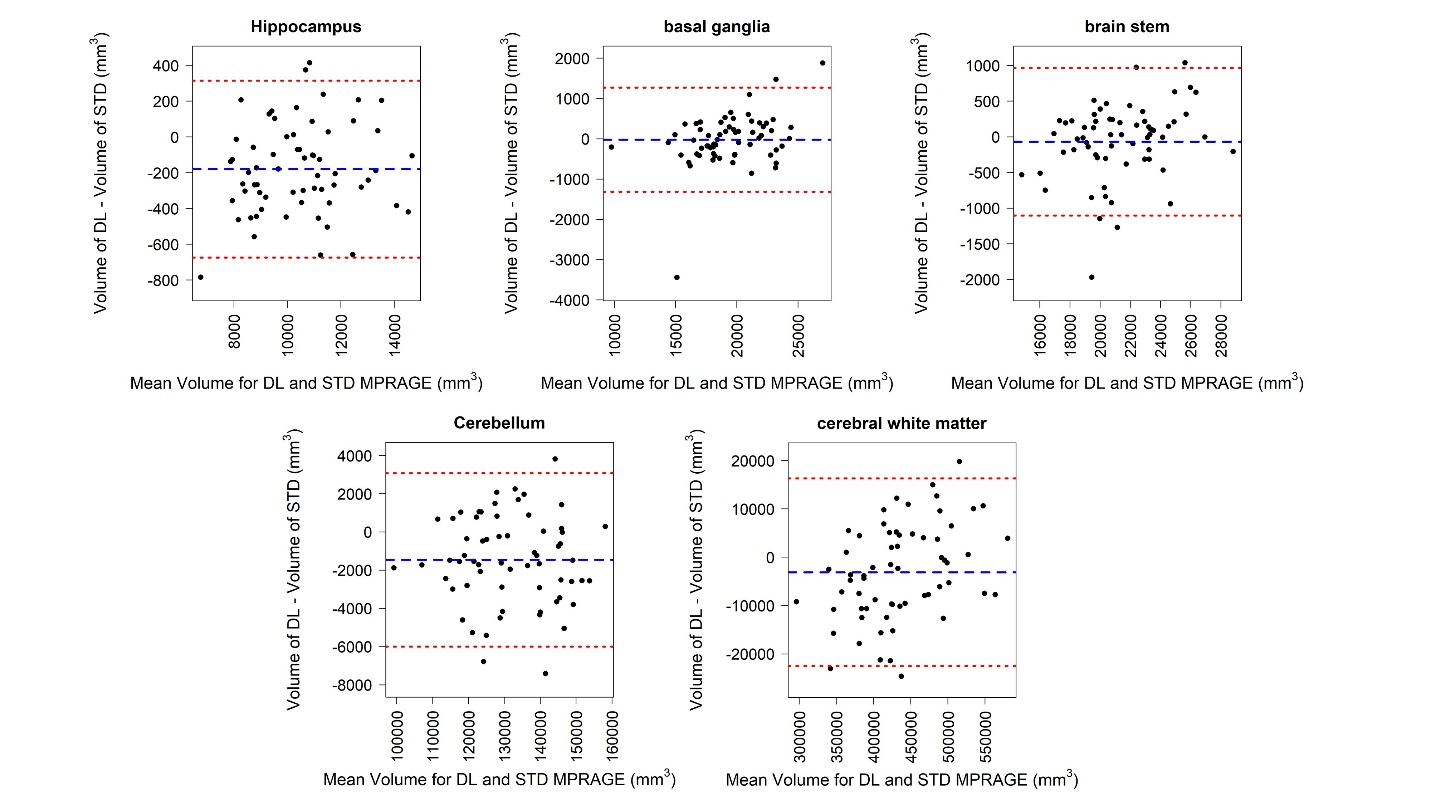


**Figure S3.** Bland-Altman analysis for the volumes of outer and inner brain structures for 63 patients (one patient was excluded due to inability to complete FreeSurfer calculations). Most data points fall within the 95% limits of agreement.


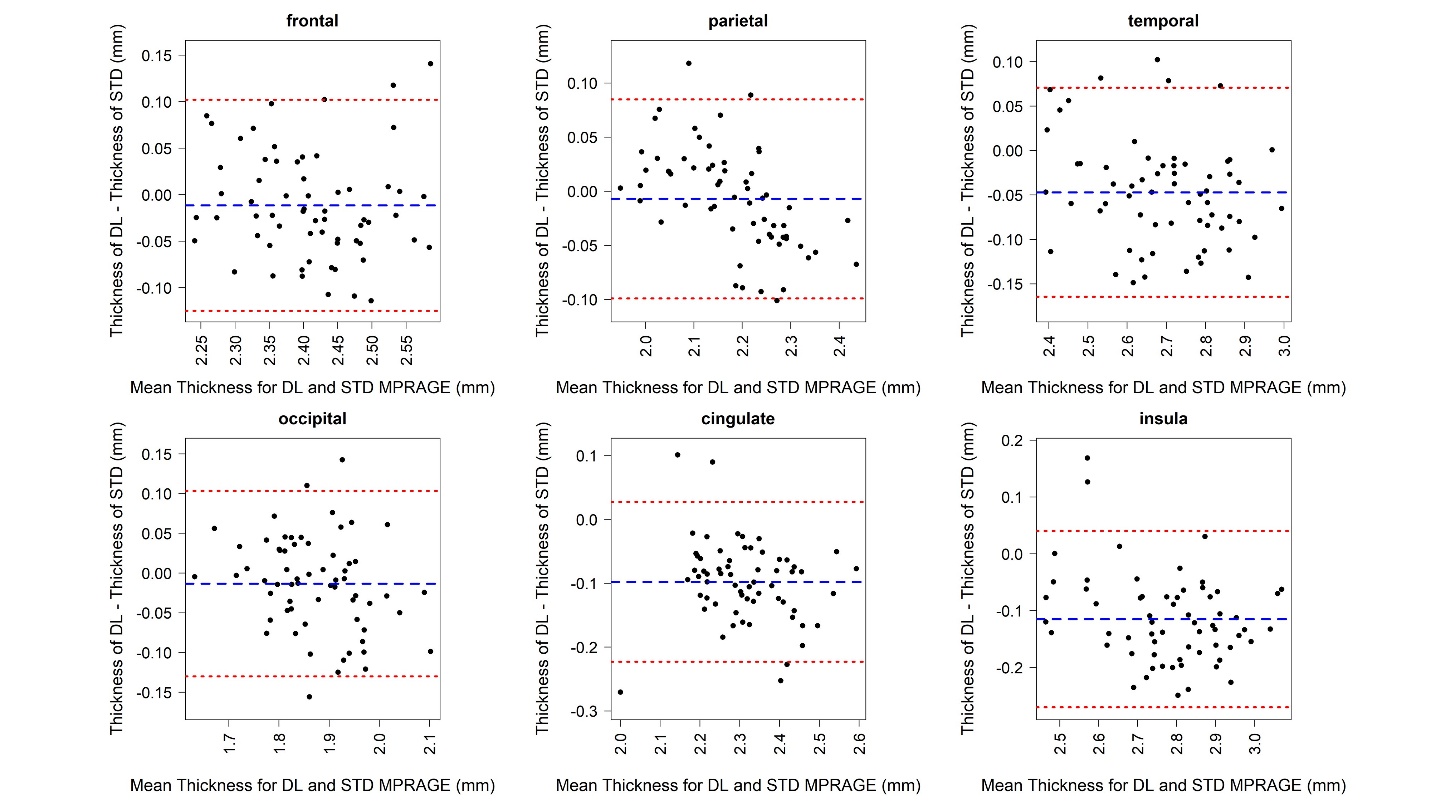


**Figure S4.** Bland-Altman analysis for the thickness of outer brain structures for 63 patients (one patient was excluded due to inability to complete FreeSurfer calculations). Most data points fall within the 95% limits of agreement.


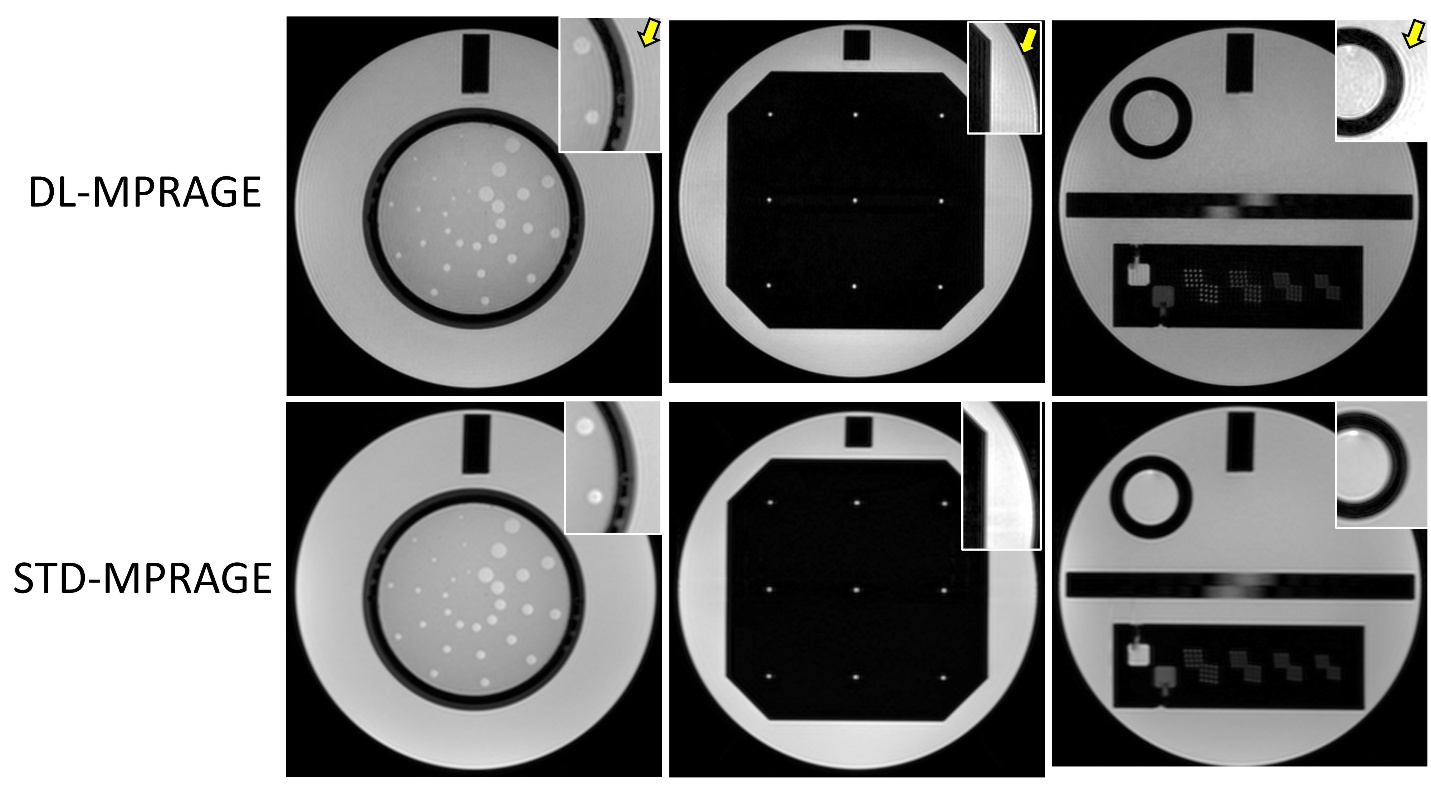


**Figure S5.** Qualitative study demonstrating three sample slices of the American College of Radiology (ACR) Phantom comparing DL-MPRAGE and STD-MPRAGE. DL-MPRAGE displays more Gibbs ringing artifacts, which tend to occur at high-contrast interfaces (top-right inlets, denoted by yellow arrows; contrast is increased from base image to highlight the artifact), but otherwise appears similar in terms of image quality.


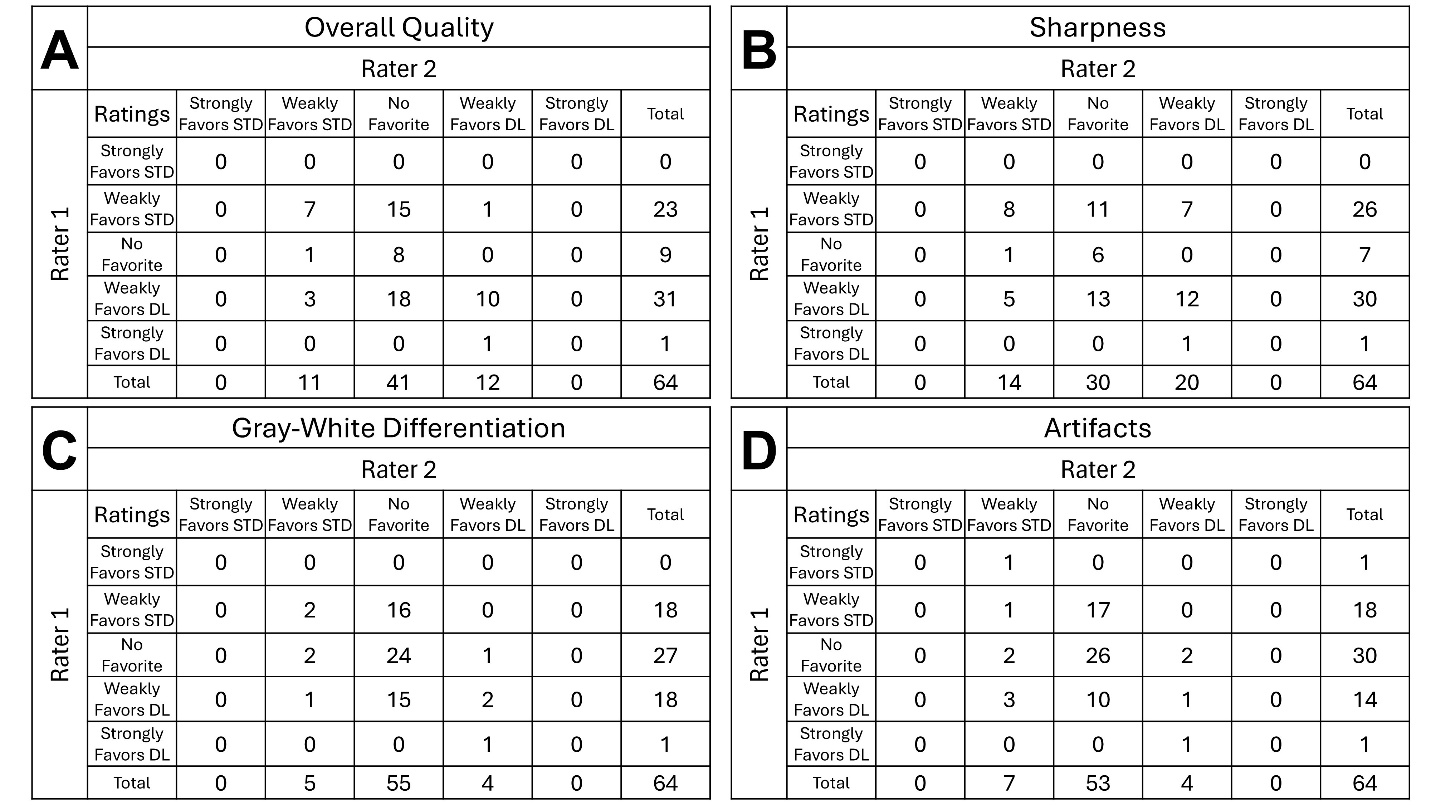


**Figure S6.** Confusion matrices describing interrater agreement for four quality measures: (A) Overall Quality, (B) Sharpness, (C) Gray-White Differentiation, and (D) Artifacts. For display purposes, “DL” represents “DL-MPRAGE” and “STD” represents “STD-MPRAGE”. “Strongly Favors STD” corresponds to qualitative ratings of -2, “Weakly Favors STD” corresponds to qualitative ratings of -1, “No Favorite” corresponds to qualitative ratings of 0, “Weakly Favors DL” corresponds to qualitative ratings of 1, and “Strongly Favors DL” corresponds to qualitative ratings of 2.


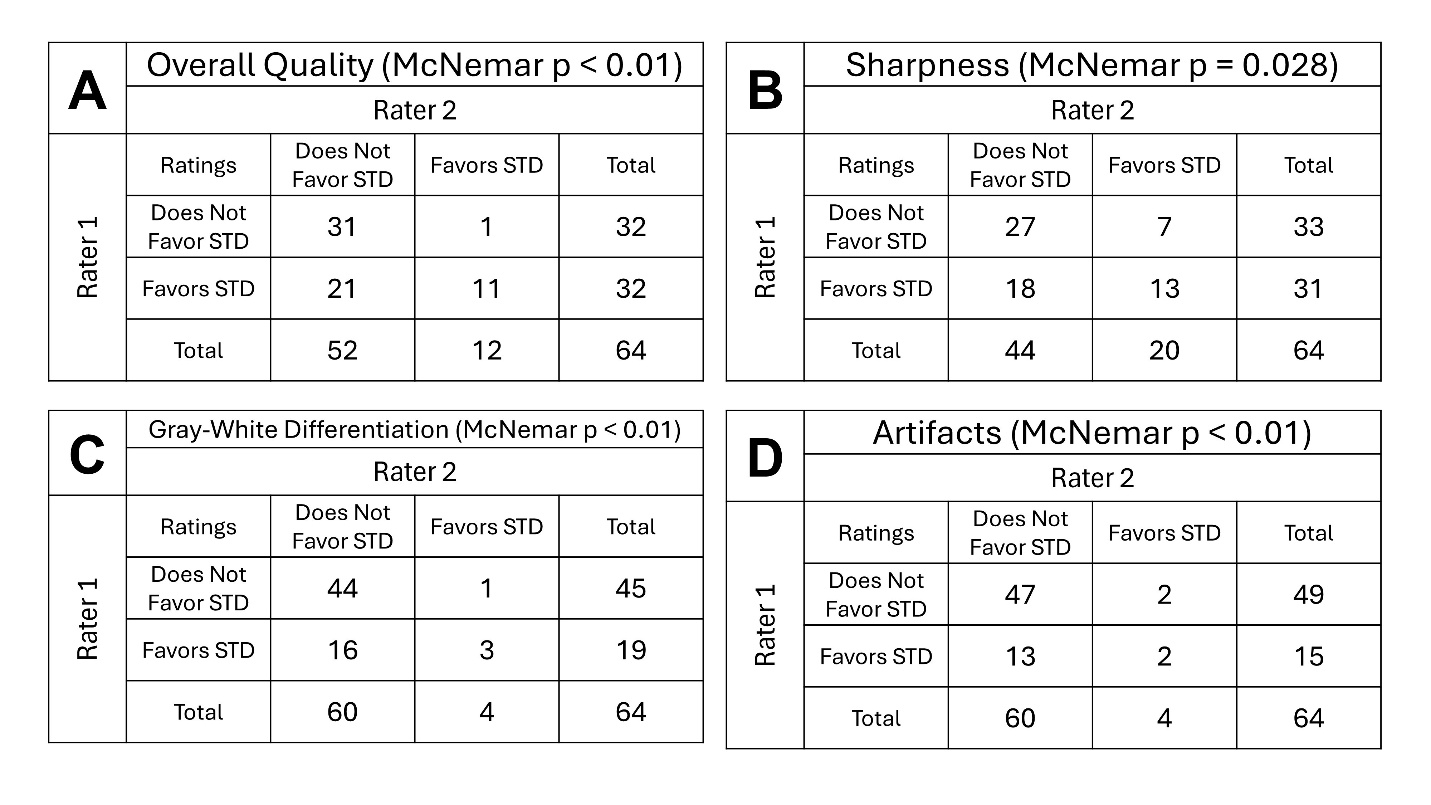


**Figure S7.** Dichotomous confusion matrices describing interrater agreement for four quality measures: (A) Overall Quality, (B) Sharpness, (C) Gray-White Differentiation, and (D) Artifacts. Rating categories are collapsed such that ratings of “-2”, “-1” are represented by “Favors STD” and ratings of “0”, “1”, and “2” are represented by “Does Not Favor STD”, with “STD” referring to “STD-MPRAGE”. McNemar test p-values are listed for each qualitative rating category.

**Table S1.** Memory Loss Protocol for our institution, including both DL-MPRAGE and STD-MPRAGE sequences.

| **Sequence** | **Duration (min:s)** |
| --- | --- |
| Standard T1 MPRAGE (STD-MPRAGE) | 5:12 |
| DL T1 MPRAGE (DL-MPRAGE) | 2:36 |
| Standard T2 SPACE FLAIR | 5:46 |
| Wave SWI | 2:08 |
| Axial GRE | 2:05 |
| Axial DWI | 1:36 |

**Abbreviations: DL-MPRAGE:** Deep-Learning Accelerated MPRAGE**, STD-MPRAGE:** Standard MPRAGE**, MPRAGE:** magnetization-prepared rapid gradient echo, **FLAIR:** Fluid Attenuation Inversion Recovery, **SPACE:** Sampling Perfection with Application optimized Contrasts using different flip-angle Evolutions, **SWI**: Susceptibility-Weighted Imaging, **GRE:** Gradient Echo, **DWI:** Diffusion Weighted Imaging

**Table S2.** Inter-reader agreement illustrated by unweighted Cohen’s kappa values for image quality ratings.

| **Feature** | **Cohen’s kappa (95% Confidence Interval)** |
| --- | --- |
| Artifacts | 0.77 (0.57 - 0.96) |
| Gray-White Differentiation | 0.91 (0.75 - 1.0) |
| Sharpness | 0.70 (0.56 - 0.84) |
| Overall Quality | 0.88 (0.77 - 0.99) |

**Supplemental Discussion**

**Interrater Agreement**

As can be seen from the confusion matrices in Figure S6, there were virtually no cases where one rater greatly favored one of the two sequences. Rater 1 tended to weakly favor either DL-MPRAGE or STD-MPRAGE, but the proportion of cases favoring either sequence was roughly equal across the four categories, with the largest difference being a slight preference for STD-MPRAGE in the Artifacts category (also corroborated by the Figure 5 balloon plot). Rater 2 most frequently regarded DL-MPRAGE and STD-MPRAGE sequences exactly equally; that rater also displayed a roughly equal proportion of cases favoring either sequence. Furthermore, regarding agreement between the raters for the dichotomized ratings, McNemar statistical testing suggested significant differences between rater classifications in three of four qualitative rating categories. This appears to be largely due to Rater 1 having more cases in the “Favors STD” category; however, it is important to interpret this in the context of the more granular data in Figure S6, where Rater 1 tended to avoid giving a “No Favorite” rating and instead chose “Weakly Favors STD” or “Weakly Favors DL” in approximately equal proportion across quality metrics. Overall, both raters still did not favor STD-MPRAGE over DL-MPRAGE for most cases, and the magnitude of disagreement is small, supporting our conclusion that DL-MPRAGE is perceived as non-inferior to STD-MPRAGE. This is well-expressed by the high Cohen’s kappa values obtained when grouping small differences in ratings that would be clinically insignificant (Supplemental Table 2).

**Generalizability of Volumetric Comparisons**

Some volumetric measurement programs, such as NeuroQuant (Cortechs.ai, San Diego, California), use normative percentiles relative to a reference population to characterize volumetric measurements of anatomical regions [4]. However, as these databases were not built using DL-accelerated MRIs, the volumetrics obtained from DL-accelerated MRIs can conceivably result in changes in normative percentiles; longitudinal analysis can be particularly affected, especially if one is interested in comparing normative percentiles on a DL-accelerated scan with those from a prior non-DL-accelerated scan. Nevertheless, data exists correlating differences in FreeSurfer-calculated volumes to normative percentile changes, which can help contextualize our study results. For example, using FreeSurfer segmentations in a normative cohort with age-adjusted regional volumetric Z-scores, a difference of 1 percentile point in left hippocampal volume corresponded to a 192 mm^3^ difference in normalized volume [5]. By comparison, the difference in hippocampal volume between DL-MPRAGE and STD-MPRAGE is estimated to be approximately 180 mm^3^, on average. Therefore, we infer that the difference in estimated volumes within the hippocampus – which is of key relevance in evaluation of Alzheimer’s disease and other dementias – is on the order of a single percentile point or less in a normative cohort.

On the other hand, one recent retrospective study of a deep-learning-based T1-weighted MPRAGE image reconstruction approach where volumetrics were obtained with NeuroQuant reported high correlations between volumes and normative percentiles of conventional and deep-learning-reconstructed examinations [6] for most evaluated anatomical regions. For example, in a validation dataset of 48 patients, a 3.7% difference in the average measured volume of the hippocampus between conventional and deep-learning-accelerated reconstructions translated to an average difference in normative percentiles of 5.9%, In the context of these results, it is unlikely that the similar differences between STD-MPRAGE and DL-MPRAGE we observed for most anatomical regions in our study would lead to large changes in normative percentiles. Nevertheless, while this prior work represents an early attempt to validation using an external test set, a more detailed assessment on a larger dataset is warranted in the future. Initial clinical validation of volumetrics obtained with deep-learning-based reconstructions such as that provided by our study represents a necessary first step before work is undertaken at a larger scale.

**Supplemental References**

[1] Wei H, Yoon JH, Jeon SK, Choi JW, Lee J, Kim JH, et al. Enhancing gadoxetic acid-enhanced liver MRI: a synergistic approach with deep learning CAIPIRINHA-VIBE and optimized fat suppression techniques. Eur Radiol. 2024.

[2] Wessling D, Herrmann J, Afat S, Nickel D, Almansour H, Keller G, et al. Application of a Deep Learning Algorithm for Combined Super-Resolution and Partial Fourier Reconstruction Including Time Reduction in T1-Weighted Precontrast and Postcontrast Gradient Echo Imaging of Abdominopelvic MR Imaging. Diagnostics (Basel). 2022;12.

[3] Fleiss JL, Cohen J, Everitt BS. Large sample standard errors of kappa and weighted kappa. Psychological Bulletin. 1969;72:323-7.

[4] Brewer JB, Magda S, Airriess C, Smith ME. Fully-automated quantification of regional brain volumes for improved detection of focal atrophy in Alzheimer disease. AJNR Am J Neuroradiol. 2009;30:578-80.

[5] Rahmani F, Jindal S, Raji CA, Wang W, Nazeri A, Perez-Carrillo GG, et al. Validity Assessment of an Automated Brain Morphometry Tool for Patients with De Novo Memory Symptoms. AJNR Am J Neuroradiol. 2023;44:261-7.

[6] Jung W, Jeong G, Kim S, Hwang I, Choi SH, Jeon YH, et al. Reliability of brain volume measures of accelerated 3D T1-weighted images with deep learning-based reconstruction. Neuroradiology. 2025;67:171-82.
